# Supplementary material for: Patterns of Intron Gain and Loss in Fungi
Source: PLoS Biol. 2004 Nov 30;2(12):e422. doi: 10.1371/journal.pbio.0020422 (PMC532390; doi:10.1371/journal.pbio.0020422)
Supplement: Table S1 — Also available at http://genes.mit.edu/NielsenEtAl/. (4.3 MB ZIP). [file pbio.0020422.st001.zip › NielsenEtAl/html/231.html]

AN2936.1.NCU07404.1.MG04464.1.FG06831.1


```
 CLUSTAL W (1.82) Multiple Sequence Alignments - Introns Inserted


Sequence 1: NCU07404.1	1089 aa
Sequence 2: MG04464.1	1073 aa
Sequence 3: AN2936.1	1059 aa
Sequence 4: FG06831.1	1136 aa
Alignment Length: 1160 aa
Number Identitical Residues: 517 aa
Alignment Score (without introns) 26913


MG04464.1 	--~------------------------------------------------------MGG
NCU07404.1	--~------------------------------------------------------MGG
FG06831.1 	MS2SINNTFDRVSSTSAVCPRHSPSPEDQLRPPVSGKQRRISNRSAPPAPGHPDIFTMGG
AN2936.1  	--~------------------------------------------------------MGG
          	                                                         ***

MG04464.1 	ESKPAAGGLKSYPLQAPKPVGKWIENIYKERLNQFKDGGQYSVQNVVG2MLEEAVAKGAP
NCU07404.1	ESATQSKPISAYPLRAPKPVGQYITNIYKDRISQFYAKGQWEHNNLNA~MMYEGVAEGEP
FG06831.1 	GTEPKGND-SSYPLFAERPVGVPKTSILKGRIEPFYKSGQYYKVNLQA2NMDNARYSGKP
AN2936.1  	DVLRQTP--SSLPRAPDGPVGQRIRSIYTDRLNQFTSRGQYQNQNLIG2KLFEATNSDED
          	         .: *  .  ***    .* . *:. *   **:   *: .  : :.  ..  

MG04464.1 	HVKLSVWDAPGQTRPKFDEATSHEFEPTDVGAWFGPAWSTHWFKIQLKIPEHMLKADLLE
NCU07404.1	HVKLFVWDAPDQDRPTFDEAVKHEFRKTEVGARFGPAWSTHWFKVVLTVPEDLRDKRLLE
FG06831.1 	HVQLSVWDAPDLSRPTFEDAVSHEFRETHTGTSFGPSWSTHWFKVILRIPEDIKDEELIE
AN2936.1  	HVKISVYSVPDLQRPSFEEATSNEFQPTHIGASFGPSWSTHWFRIRLTIPEDMRERERLE
          	**:: *:..*.  **.*::*..:**. *. *: ***:******:: * :**.: .   :*

MG04464.1 	FHWNAGNEGMVWTAEGNPIQGLSEQ-QREEWVIPDSFRDGKEHIIYIEMACNGMFGAAPG
NCU07404.1	LHWDANNEGMVWTHDGKPLQGLTGGGERTEWILPDSFRDGKEHIIYIEMACNGMFGAAGG
FG06831.1 	LHWDASNEGTIWTEDGVPIQGLTGSGERIEWIIPDSFRDGEEHTIYIEMACNGMFGNAPN
AN2936.1  	FHWDANNEGLVWTEDGHPLQGLTGGGERIEWIIPDAWRDGNEHVFYIEMACNGMFGNAPG
          	:**:*.*** :** :* *:***:  .:* **::**::***:** :*********** * .

MG04464.1 	--DSIQPPDPNKYFRLGKAEIVAVNPEARQLIIDHWIIGD~AAREFPQDSWEQHKALKVA
NCU07404.1	G-DIIQPPDPNKHFRLDKAEIVAVNPDARQLYIDIWIIGD~AAREFPSESWEQHQALKTC
FG06831.1 	GTTTIAPPDPNRRFNLSKADIVAVNVPARKLHIDIWEIGD~AARELPENSAEQNHALAVA
AN2936.1  	G-DSIQPPAPDKYYTLTTARITAVNLEARALFYDFWIISD1AAREFPAEGWESHEANMVA
          	.   * ** *:: : * .* *.***  ** *  * * *.* ****:* :. *.:.*  ..

MG04464.1 	TDMMEAFEFGNK--ESIVKARKIAREYLG-DVNSHKVYDSE--KEALVYGIGHCHIDTCW
NCU07404.1	NEIIEAFELGNK--DSLKKCREIAQQYLGPDVDSARVYDTKRGKAPLVFGIGHCHIDSCW
FG06831.1 	MKIINTFEVNNQ--DSILKCREIAREILGPDVDSHKVYEVG--KDPVVFGIGHCHIDSCW
AN2936.1  	NSIIDAFIAGNGSLESIKEGRRIAQKYLGDKVDCSEVYDSG--TQPIVYGIGHCHIDTCW
          	 .::::*  .* : :*: : *.**:: ** .*:. .**:    . .:*:********:**

MG04464.1 	LWPWAETRRKTARSWSNQCDLMDRYPELHFAVSQAQQYKWLKEDYPYVFDRIKKKIKDGR
NCU07404.1	LWPWDETKRKVARSWSNQCDLMDRYPELNFACSQAQQYKWCKTLYPHVWERVKSKVADGR
FG06831.1 	LWPWAETKRKVVRSWMNQCDLMERYPEANFACSQAQQYKWLKTYYPAAYKRVKQKVKEGQ
AN2936.1  	LWPWAETKRKVARSWSNQCDLMDRYPEHRFTCSQAQQFKWLKQYYPSVFDRVKGWVKKGH
          	**** **:**..*** ******:**** .*: *****:** *  ** .:.*:*  : .*:

MG04464.1 	FHPIGGSWVEHDTNMPSGESLVRQFLYGQRFFEGNFGKRCQTFWLPDTFGYSSQLPQICR
NCU07404.1	FHPIGGSWVEHDTNMPSGESLVRQFVYGQRFFESNFGKRCQTFWLPDTFGYSSQLPQLCR
FG06831.1 	FHPIGGSWVEHDTNLPSGESLVRQFFYGQRFFEAEFGSRCRTFWLPDTFGYSSQLPQLCR
AN2936.1  	FQPIGGSWVEHDTNMPSGESLVRQFIYGQRFFESNFGERCTTFWLPDTFGYSTQIPQLCR
          	*:************:**********.*******.:**.** ***********:*:**:**

MG04464.1 	LAGMNRFLTQKLSWNNINKFPHTTFNWVALDGTQVICHMPPSETYTAGANFGDVKRSITQ
NCU07404.1	LAGMTRFLTQKLSWNNINKFPHTTFNWVALDGTQVICHMPPSETYTAEAHFGDVKRSMSQ
FG06831.1 	LADMDRFLTQKLSWNNINNFPHTTFMWVSPDGSQVICHMPPSETYTANADFGDLKRSIEK
AN2936.1  	LAGMSRFFTQKLSWNNINNFPHTTFQWVALDGSQVMCHMPPSETYTAEAHFGDLKRSITQ
          	**.* **:**********:****** **: **:**:*********** *.***:***: :

MG04464.1 	HKSMNQDETSLLVFGHGDGGGGPTWEHLEKLRRCRGISDKVGLLPRIHLGNTVDDFFDKL
NCU07404.1	HKSLDQDHTSLLVFGKGDGGGGPTWQHLEKLRRCRGISDTVGLLPRVSLGGSVDDFFDSL
FG06831.1 	HKTMRVDSSSLLVFGKGDGGGGPTWQHFEKLRRCAGISNTIGGIPKIKMGLTVDDYFDRL
AN2936.1  	HKSLDKDNTSLLVFGKGDGGGGPTFEHLEKLRRCRGLSDKTGLLPRATMGTSVDDFFAKL
          	**::  * :******:********::*:****** *:*:. * :*:  :* :***:*  *

MG04464.1 	QNKADD---FVTWYGELYFELHRGTYTTQANNKLNNRKSETVLRDLEFLATIATIKDNKY
NCU07404.1	ERKADS---LVTWFGELYFELHRGTYTTQANNKKFNRKAEFVLRDIEQLATIASLKDKDY
FG06831.1 	NQKATE---FPTWYGELYFELHRGTYTTQANNKYYNRKAEVMLRDIEQLATFASIKNKKY
AN2936.1  	EKKAADGTKFATWYGELYFELHRGTYTTQANNKRNNRRAEFLLRELEFLATIASITRSDY
          	:.** ..:.: **:*******************  **::* :**::* ***:*::. ..*

MG04464.1 	KYPKKEIDDMWEWTLLCQFHDCLPGSSIEMCYDDSDE0LYAKLFETAARVRKEIYDVLGL
NCU07404.1	KYPKKEIDEMWEATLLCQFHDCLPGSSIEMCYDDSDK0LYAQIFKTGERLLRDACKALGV
FG06831.1 	KYPTKDLDDMWESVLLCQFHDCLPGSSIEMCYDDSDK0VYAEVFETGKRLLNDLYDALHV
AN2936.1  	EYPKKDIDEMWEGVLLCQFHDCLPGSSIEMCYDDSDE~LYAQIFETGLRAREAALEALGF
          	:**.*::*:*** .**********************: :**::*:*. *  .   ..* .

MG04464.1 	SDVKATSIQSAAALNTLPWHRKEIIDINEDEGAVACGSGNILH-----VHPFKTSEKKQ-
NCU07404.1	FQVDATGVERGIALNTLPWYREELVEISKGDFALAKGVGPLLK-----LQQYEIMQVDDG
FG06831.1 	TSQFSSSLDESVAINTLPWHRKELVELSDSEVGVACGDGQLLA-----LRSFKVQEEKPA
AN2936.1  	SMAG----RVPVAINTLPWNRSEVVRLP-ADMATNGSMHALLNGGPGVIPHSQAIARVPE
          	            *:***** *.*:: :   : .   .   :* .... :   :       

MG04464.1 	VSVSKEDDGVFIMENDDLKIKVKNGVITSMYDRRVGREVIPKGGKANQFVIFDDKPLYWQ
NCU07404.1	VKLHKLSNGVFVLENKRLKVRVEAGTITSLYDKYDRREVIPEGQRANQFVIFDDKPLYWQ
FG06831.1 	VTVMEQSTDVYVLQNDQLRVVVDNGVITSIYDIENDREVVEKGGEANKFVIFDDIPLYWQ
AN2936.1  	VSVTEIKPGVFRLNNGKLRVDVEDGLITSLYDIEADREVIAADGRAGQLVIFDDKPLYWQ
          	*.: : . .*: ::*  *:: *. * ***:**    ***:  . .*.::***** *****

MG04464.1 	AWDVEVYHLDSRKELTSGTTEISENKAHRVSVVTKTKISDNSSIKTTISLSAAIQGQQSH
NCU07404.1	AWDVEVYHLDTRKELQSSETQVHEEKTHRVSVITKTKISEQSHISTIISLSAAVNELQSY
FG06831.1 	AWDVEVYHLDARRNVEYGKTKIFEQKPHRVTLVTEIKVSENSSIKSYTTLSAALKGQPPQ
AN2936.1  	AWDVEVFHLDSRKELQSGKTTIAENDPYRVSVVTETKISEKSWVKTTISLAATEGNRPSY
          	******:***:*:::  . * : *:..:**:::*: *:*::* :.:  :*:*:     . 

MG04464.1 	VEVHSEVDWHETMKFLKVEFPVDIRNTEASYETQFGIVKRPTHYNTT~WDMAKFEVCCHR
NCU07404.1	IEVQSTVEWHETMKFLKVEFPVDIRNTEASYETQFGIVRRPTHYNTS~WDMAKFEVCCHK
FG06831.1 	IDVKANVNWHEDSKFLKVEFPVNVVNNEASYETAFSITKRPTHYNTS~WDMAKFEVCCHK
AN2936.1  	VELESEVEWQETMKFLKVEFPVDITNTEASYETQYGIIRRPTHYNTS2WDMAKFEVCAHK
          	:::.: *:*:*  *********:: *.****** :.* :*******: *********.*:

MG04464.1 	FADLSENGYGVSILNDSKYGFATVGNLMRLSLLRAPKAPDAHADMGKHHIRWAILPHEGA
NCU07404.1	FADLSEHGYGVSILNDSKYGFATTGSLMRLSLLRSPKAPDAHADMGAHTIRWAIFPHTGS
FG06831.1 	FADLSENNYGVSILNDCKYGFATAGKMMRLSLLRSPKAPDANADMGRHTIRWAILPHKGG
AN2936.1  	WADLSENGYGVSILNDSKYGFATCGNLMRLSLLRAPKAPDAHADMGRHTIRYAIFPHSGP
          	:*****:.********.****** *.:*******:******:**** * **:**:** * 

MG04464.1 	LGHTTVHRAIEFNNKLTV---LSKPADEAAAMLSSCPIKVTGN--PSLVLDTVKRGEDDE
NCU07404.1	LGAMTVRKAYEFNNPLKL---LESPHSFATLDEQEPPVWFTDNSNPSLILDTIKRAEDDE
FG06831.1 	LSSTTVKAAYAFNNPLKP---VTASKVVLESLSSAPIKLVNTDESDSLVLDTIKRGQDDE
AN2936.1  	LDFRTVRAGYEFNHPLILDTSLALPDEQSVFAVSALFRKIELTGSPSLILDVVKRGEDDE
          	*.  **: .  **: *  .:::  .        .     .    . **:**.:**.:***

MG04464.1 	DVSVG-LLP----ARKGRSVIVRIYDSLGGQARGAVETSWD~V~KKVRKVNILEDDGDE-
NCU07404.1	DVSVE-TIESPVKKKKGQSVILRIYDSLGGRARGTIETRWP~V~KRVSKVNLLEDELEEV
FG06831.1 	DVTRKEGLR----VNKGQSIIIRVYESLGGRSRGTIKTSFD~V~KRVTKTNILEDDLEE-
AN2936.1  	DIDGDPDISLPSSKRSGQSIILRIYESLGGKSRGAIRTALP0F2----------------
          	*:     :  . . ..*:*:*:*:*:****::**::.*    .                 

MG04464.1 	--LEFKDGKFEIELRAFEVATFRLEL-
NCU07404.1	SGFDVAEGRFEVDLRPFEVASYRLELA
FG06831.1 	--IEYEDGKIPITLRPFEVATFKLQL-
AN2936.1  	--FIAWSGMF-----------------
          	  :   .* :
```
